# Machine learning based model for the early detection of Gestational Diabetes Mellitus

**DOI:** 10.1186/s12911-025-02947-3

**Published:** 2025-03-13

**Authors:** Hesham Zaky, Eleni Fthenou, Luma Srour, Thomas Farrell, Mohammed Bashir, Nady El Hajj, Tanvir Alam

**Affiliations:** 1 College of Science and Engineering, Hamad Bin Khalifa University, Doha, Qatar; 2 Qatar Foundation for Education, Science, and Community, Qatar Biobank for Medical, ResearchDoha, Qatar; 3 College of Health and Life Sciences, Hamad Bin Khalifa University, Doha, Qatar; 4 Endocrine Section, Department of Medicine, Hamad Medical Corporation, Doha, Qatar; 5 Qatar Metabolic Institute, Hamad Medical Corporation, Doha, Qatar

**Keywords:** Gestational Diabetes, Machine Learning, Qatar Biobank (QBB)

## Abstract

**Background:**

Gestational Diabetes Mellitus (GDM) is one of the most common medical complications during pregnancy. In the Gulf region, the prevalence of GDM is higher than in other parts of the world. Thus, there is a need for the early detection of GDM to avoid critical health conditions in newborns and post-pregnancy complexities of mothers.

**Methods:**

In this article, we propose a machine learning (ML)-based techniques for early detection of GDM. For this purpose, we considered clinical measurements taken during the first trimester to predict the onset of GDM in the second trimester.

**Results:**

The proposed ensemble-based model achieved high accuracy in predicting the onset of GDM with around 89% accuracy using only the first trimester data. We confirmed biomarkers, i.e., a history of high glucose level/diabetes, insulin and cholesterol, which align with the previous studies. Moreover, we proposed potential novel biomarkers such as HbA1C %, Glucose, MCH, NT pro-BNP, HOMA-IR- (22.5 Scale), HOMA-IR- (405 Scale), Magnesium, Uric Acid. C-Peptide, Triglyceride, Urea, Chloride, Fibrinogen, MCHC, ALT, family history of Diabetes, Vit B12, TSH, Potassium, Alk Phos, FT4, Homocysteine Plasma LC-MSMS, Monocyte Auto.

**Conclusion:**

We believe our findings will complement the current clinical practice of GDM diagnosis at an early stage of pregnancy, leading toward minimizing its burden on the healthcare system.Source code is available in GitHub at: https://github.com/H-Zaky/GD.git

## Introduction

Gestational diabetes mellitus (GDM) is a significant health challenge affecting over 14% of pregnancies worldwide [1]. In Qatar, GDM is high in prevalence, with an incidence of 23.5% across all pregnancies [2, 3]. GDM is a form of hyperglycemia characterized by increased insulin resistance arising during the second trimester of pregnancy [4]. GDM is defined as any form of hyperglycemia that is first detected during pregnancy. Insulin resistance and relative insulin deficiency are the main causes of GDM. Insulin resistance increases gradually by mid-gestation, secondary to the rise in placental hormones such as human placental lactogen and cortisol. Therefore, pancreatic ß cells increase insulin production to oppose the desensitizing effects of placental hormones and retain blood sugar levels within the normal range [5]. In GDM pregnancies, pancreatic ß cell dysfunction prevents glucose level normalization, causing maternal and fetal hyperglycemia [6]. GDM is associated with several pregnancy complications, such as large for gestational age, macrosomia [7], pre-eclampsia, pre-term deliveries and increased rates of C-section. Women who develop GDM during pregnancy are at a high risk of developing Type 2 diabetes (T2D) [8, 9]. Furthermore, infants born to mothers with GDM have a higher lifelong risk of metabolic disorders [10]. As such, GDM is considered a critical factor in the rising incidence of T2D and obesity globally. The risk factors of GDM include a family history of diabetes, age, low physical activity, high pre-pregnancy body mass index (BMI), and poor dietary habits [11–13].

Gestational diabetes is often diagnosed between 24-28 weeks of gestation using an Oral Glucose Tolerance Test (OGTT) [14–16]. Therefore, there is an urgent need to develop strategies for early detection of GDM to avoid potential complications and late diagnosis. State-of-art deep learning techniques are integrated with a novel multi-scale feature extraction approach to enable precise and efficient GDM detection. Our model has an innovative structure and algorithmic enhancements that aim to overcome the drawbacks of existing approaches, resulting in a robust solution for clinical use. This would allow early monitoring and intervention for women at risk of GDM, thus minimizing adverse outcomes for both mothers and offspring.

The contribution of this work can be summarized as follows:

1. This is the very first study in Qatar for the early prediction of GDM based on Machine Learning (ML) models using first-trimester clinical data only.
2. We propose a stacking-based ML model that achieved 88.8% accuracy in detecting GDM from the control group.
3. We show that homeostasis model assessment insulin resistance (HOMA-IR) score, Insulin and history of diabetes are the most prominent attributes along with Uric Acid, Cholesterol, Urea, prothrombin time for the early detection of GDM.

### Background Studies

Xiong et al. conducted a study on predicting GDM based on 215 patients and 275 controls for the prediction of GDM in the first 19 weeks of pregnancy [17]. The proposed support vector machine (SVM) based model using prothrombin time and activated partial thromboplastin time achieved 88.3% sensitivity and 99.47% specificity. Moreover, using renal and hepatic function, the proposed model achieved 82.6% sensitivity and 90% specificity. Zhang et al. used ultrasound and serological markers from 1000 patients collected during 24-28 weeks of pregnancy for GDM detection [18]. Their proposed logistic regression-based model achieved 83% sensitivity and 83% accuracy. Zhang et al. [19] performed a meta-analysis on 25 studies using machine learning based models for predicting GDM. The study highlights the accuracy of ML methods in predicting GDM and the highly contributing features used in the model, including maternal age, family history of diabetes, BMI, and fasting blood glucose. Current GDM screening tests are performed later in pregnancy, potentially overlooking opportunities for early intervention through diet or exercise that can significantly benefit maternal and child health. In this study, we considered a ML approach for early GDM detection using clinical markers collected during the first trimester. The data were collected before the 12th week of pregnancy as part of the Qatar Birth Cohort Study (QBiC). Our model achieved high accuracy in detecting GDM from the control group using only the first trimester data. A brief summary of the previous work that used ML for GDM is highlighted in Table 1.

**Table 1:** Summary of existing literature that employed ML models for GD

Reference	Cohort	Models used	Results
[19]	Sample sizes varied from 134 to 66,687, China	Logistic regression (LR), SVM, Bayesian, and Ensemble methods	Non-LR models AUROC: 0.8891
[20]	Internal Cohort: 1148, United Kingdom External Cohort: 709 patients, United Kingdom	XGBoost regression model	Internal: MSE: 0.021 External: MSE: 0.02
[21]	Pregnant women at risk of GDM, China	Ensemble learning algorithm with XGBoost, LightGBM, and CatBoost models	Accuracy: 80.3%, Precision: 74.6%, Recall Rate: 79.3%
[22]	34,387 pregnancies, South Korea	XGBoost	AUC values of 0.804 at M1, 0.721 at E1, 0.720 at E0, and 0.711 at baseline in the whole cohort
[23]	19,331 pregnancies, China	XGBoost	AUC (0.742, p *<*0.001)
[24]	7,594 pregnancies included from XHCM and SPNPH, China	Logistic Regression, XGBoost, and two ensemble algorithms	XHCM: AUC = 0.99 SPNPH: AUC = 0.83
[25]	1,611 pregnancies, Chile	Gaussian Na¨ıve Bayes, Decision Trees, Support Vector Machines, and others	AUCROC: 0.81–0.82
[26]	67 pregnant women, China	Machine learning ensemble model	AUC of ROC: 0.81 (training), 0.71 (testing)
[27]	909 pregnancies, Singapore	CatBoost	AUC: 0.85
[28]	484 pregnant women from the PEARS study, Ireland	SVM-based models (Model 1, Model 2, Model 3)	Model 1: AUC-ROC: 0.792 Model 2: AUC-ROC: 0.659 Model 3: AUC-ROC: 0.656
[29]	1,443 pregnant women, South Korea	Logistic regression, random forest, support vector machine, and deep neural networks	AUC: 0.740-0.781
[30]	925 pregnant women, China	XGBoost, Logistic Regression (LR)	XGBoost: AUC: 0.946 LR Model: AUC: 0.752
[31]	82,698 pregnancies, Japan	Random Forest (RF), Gradient Boosting Decision Tree (GBDT), Support Vector Machine (SVM), and Logistic Regression (LR)	GBDT for GDM-PH(+) group: AUC=0.67 GBDT for GDM-PH() group: AUC=0.74
[17]	490 pregnant women in the first 19 weeks of pregnancy, China	SVM, LightGBM	PAT-PT and PAT-APTT: AUC: 94.2% DBIL and FPG: AUC: 91.0%
[32]	30,474 pregnancies, Northern California	CART, LASSO regression, and Super Learner (SL) with RF and XGBoost	AUC: 0.934
[33]	48,502 singleton pregnancies, Australia	CatBoost and XGBoost alongside logistic regression	Accuracy: 85% F1-score: 84%
[34]	1075 pregnant women, China	Score-Scaled Model, Logistic Regression Model, Decision Tree (DT) Model, Random Forest (RF) Model	Score-Scaled Model: AUC of 0.772 LR Model: AUC: 0.799 DT Model: AUC: 0.825 RF Model: AUC: 0.823
[35]	1000 samples, China	Random Forest (RF), Logistic Regression	AUC: 82.5%
[36]	Pregnant women, South Africa	DT and RF Regressors, Coupled-Matrix Tensor Factorization, and Elastic Net techniques	MSE: 0.29–0.42
[37]	17,005 pregnant women, China	Logistic Regression (LR), Random Forest (RF)	AUC: 0.746
[38]	1,139 pregnant women, China (2017-2019)	Random Forest and Logistic Regression	RF Results: AUC: 0.777 ± 0.034 Logistic Regression: AUC: 0.755 ± 0.032

## Materials and methods

**Fig 1.**
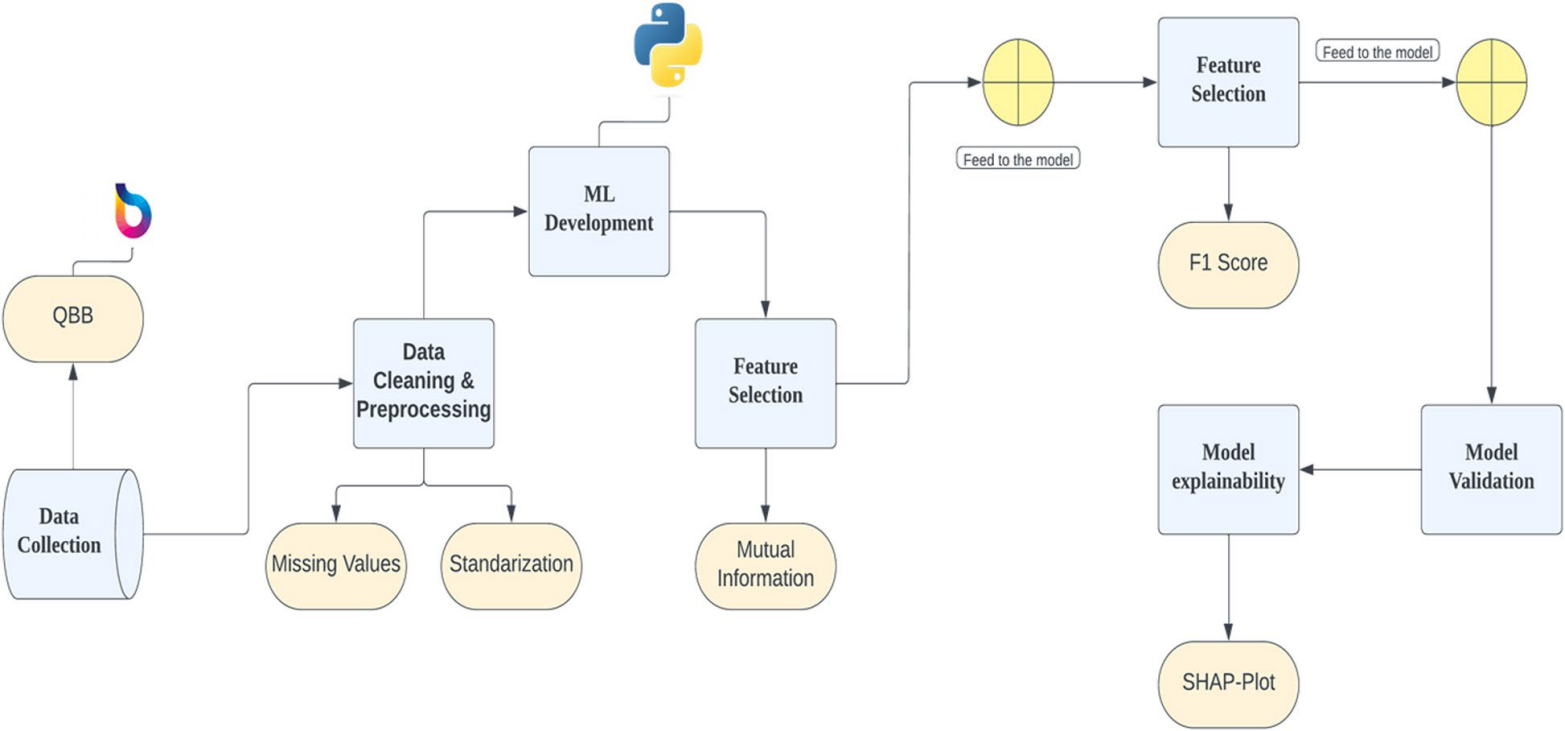
Schematic Diagram of the Workflow

In our study, we started by collecting the data and selecting the top features using the feature selection phase. Next, we used Mutual Information (MI) and F1-score based methods for further feature engineering. Afterwards, we developed a Machine learning (ML) model, incorporating model validation through random seeds and cross-validation. Statistical analyses are employed to achieve model explainability, ensuring transparency and reliability in decision-making. In addition, feature importance is visualized using SHAP values. A summary of the overall workflow is highlighted in Figure 1.

### Data collection and description

The dataset used in this study comprises first-trimester data collected from a cohort of 138 female patients who were under observation at Hamad Medical Corporation (HMC). Then, during the second trimester, the same group of patients revisited HMC for the second data collection phase. Among the 138 pregnant women included in this study, 63 women were diagnosed with GDM in the second trimester, and 75 women were GDM-free. Features from the first-trimester data were employed for the early detection of GDM, whereas GDM onset as class label was incorporated from the second-trimester dataset collected on the same patients. A rich set of 68 distinct features has been meticulously curated Within each patient’s dataset. Along with Absolute Neutrophil count (ANC), features had a broad spectrum of hematological parameters as well as physiological and biochemical variables such as Basophil count, Eosinophil count, Hematocrit (Hct), Hemoglobin (Hgb), and various other components that contribute to a comprehensive understanding of the patient’s health status. Not only traditional blood cell counts and blood chemistry markers were covered by the dataset’s extensive feature set, but they also included advanced biomarkers such as NT pro-BNP, a marker for heart-related conditions, and a panel of metabolic indicators like cholesterol, glucose, and triglyceride levels. Additionally, the dataset included markers related to liver function (e.g., ALT, AST, Alk Phos), renal function (e.g., Creatinine, Urea), and various hormonal markers, offering a holistic view of the patient’s physiological state. All 68 features are mentioned in Table 2.

**Table 2:** List of all 68 features available in our dataset

Absolute Neutrophil Count Auto# (ANC)	Basophil Auto #	Basophil Auto %	Eosinophil Auto #	Eosinophil Auto %	Hct
MCHch	MCHCchc	Mcv	Monocyte Auto #	Monocyte Auto %	Mpv
Wbc	Nt Pro-Bnp	Albumin Lvl	Alk Phos	Alt	Ast
Calcium Corr	Chloride	Cholesterol	Ck	Creatinine	Ggt
Ldl-Calc	Magnesium	Phosphorus	Potassium	Sodium	Tibc
Uric Acid	Aptt	Fibrinogen	Prothrombin Time	C-Peptide	Estradiol
Ft4	Insulin	Testo	Tsh	Vit B12	Vit D
Hgb	Lymphocyte Auto #	Lymphocyte Auto %	Neutrophil Auto %	Platelet	RBCbc
Bicarbonate	Bilirubin T	Calcium	Glucose	HDLdl	Iron
Total Protein	Triglyceride	Urea	Ferritin	Folate	Ft3
Crp	Hba1c %	Homocysteine Plasma Lc-Msms	HOMA-IR 405 Scale	History Of High-Level Glucose Or Diabetes	Weight Difference
Family History Of Diabetes	HOMA-IR 22.5 Scale				

### Data cleaning and pre-processing

This study carried out a series of pretesting steps to acquire the dataset for data quality guarantee. Using Python, the data stored in an Excel file was initially loaded into a Pandas DataFrame using a specified file path. Missing data analysis was then conducted within each class defined by ”Case” or ”Control,” which revealed the patterns in these classes to ensure dataset integrity and reliability. In addition, a methodical approach called median imputation has been used to address the missing values of a given class according to their specific characteristics. The difference between ”case” and ”control” classes were addressed in allocating missing values.

The specificities of each class have been preserved, using the pandas library to perform this imputation by calculating medians in their various groups. Reading the data set in an Excel file, determining its shape, and determining which columns do not contain any values started this process. Subsequently, the missing data were analyzed in class distribution using group operation to identify absence patterns within individual classes. Using the ’group by’ and ’transform’ functions, the key steps were calculating each class’s median values. This class-specific approach ensured that imputed values retained the statistical characteristics of their respective classes. Following this calculation, the missing values in the dataset were replaced by the calculated median values using the fill method, which improved the completeness of the dataset. Finally, a new Excel file has been saved to the resulting preprocessed dataset, which now contains no missing values due to the successful median imputation.

### Features normalization

In this stage, we normalized the features using following equation.

$$z=\frac{X-U}{S}$$

Where:

- Z is scaled data
- X is the data point
- U is the mean of the training samples
- S is the standard deviation of the training samples.

’StandardScaler’ was used to transform the features into a standardized distribution characterized by a mean of 0 and a standard deviation of 1 using python. This step was conducted to ensure a consistent scale across all variables.

### Features subset selection

We used mutual information (MI) based feature ranking to select a subset of features. The degree of information sharing between each feature and the target variable is measured quantitatively by the MI score. The methodology reveals the intrinsic relationships that lead to the predictive power of each feature through its analysis of these common dependencies. As shown in the formula below, the mutual information between two random variables, X and Y, may be formally indicated.

$$I\left(X;Y\right)=H\left(X\right)-H\left(X|Y\right)$$

Where:

- I(X; Y) is the mutual information for X and Y
- H(X) is the entropy for X, and H(X — Y) is the conditional entropy for X given Y.

Features having higher score than zero were kept after the MI scores were calculated and arranged in descending order. Variables that had low scores which indicated their low predictive power were eliminated. Features that had scores higher than zero were kept after the MI scores were calculated and arranged in descending order. Variable The F1 score-based filtering technique we employed assessed each feature’s contribution to the precision and recall of the model, further ensuring robust feature selection. This two-step selection procedure helps to increase the accuracy and interpretability of the model by removing superfluous or unnecessary features.

### Machine learning modelling

ML models have been widely used in the early detection of multiple diseases [39, 40]. For the early detection of GDM, features were obtained from first-trimester data and GDM onset as class label was obtained from the second trimester dataset of the same longitudinally followed patients. In constructing the ML models, different models as well as ensemble of the models were used: (a) Random Forest Classifier, (b) Gradient Boosting Classifier, (c) AdaBoost Classifier, (d) Decision Tree, (e) Logistic Regression, (f) Support Vector Classifier, (g) GaussianNB, (h) KNeighbors Classifier, (i) CatBoost Classifier, (j) XGB Classifier, and (k) LGBM Classifier as a base model. For the ensemble model, all these eleven models were combined using “StackEnsemble” in python, and Logistic Regression Classifier was employed as the Meta model.

A. The Random Forest Classifier has been included because of its ability to handlecomplex datasets and capturing non linear relationships, which gives a solid foundation. Complementing this was the selection of GradientBooster and AdaBooster Classifiers for improved total accuracy over multiple iterations, which is a useful tool to seek out complex patterns in your data.
B. Decision trees inherently reveal the decision-making process that’s why we in-cluded Decision Tree Classifier as it aligns with the aim of incorporating interpretability into the model. This is especially crucial in medical diagnostics where interpretability is a significant consideration.
C. The classical Logistic Regression was integrated for its simplicity and inter-pretability, serving as a baseline model, and effectively capturing linear relationships within the dataset.
D. The Support Vector Classifiers SVC is considered appropriate to capture complexrelationships in High Dimensional spaces. It is an excellent addition, especially in cases where complex patterns can be observed, because of its ability to determine optimal hyperplanes for the division of classes.
E. The Gaussian Naive Bayes model, known for its simplicity and efficiency, wasincluded, leveraging the assumption of feature independence.
F. The KNearest Neighbors model, based on the majority class of their neighbors,has been developed using a proximity based approach for classifying data points. It is well suited for the identification of localised patterns, which can have a decisive effect in diagnosis of diabetes during pregnancy.
G. In view of the nature of medical datasets, CatBoost Classifier has been selectedfor its ability to efficiently control categorical features. Finally, the XGBoost and LightGBM classifiers, which are known to be effective and efficient in handling complex datasets, have been integrated.

These models contribute to the overall model’s ability to predict, bringing a degree of sophistication into the ensemble. Collectively, the diverse set of base models aims to provide a comprehensive and accurate framework for gestational diabetes detection, leveraging the strengths of each algorithm to collectively enhance the model’s predictive power.

 Using a pool of Random Seed for the generalization capability of ML models Our machine learning model was evaluated using a collection of random seeds to ensure its robustness and reproducibility. Given the 138 patients in the cohort, we devised a pool of 50 random seeds to handle data uncertainty. Performance metrics were aggregated across multiple iterations to provide an unbiased evaluation of the model after initialization. Reducing the impact of random fluctuations in the data, this approach aids in assessing the model’s stability and dependability. The model’s sensitivity to initial conditions was evaluated by systematically varying the random seeds, which ensured that the reported performance metrics were robust and not artifacts of specific data splits. The use of random seeds also enhances the reproducibility of our experiments, as other researchers can replicate our results by using the same seed values. This also enhances the reproducibility of our experiments, as other researchers can replicate our results by using the same seed values.

## Results

### Baseline statistics

We had a total of 138 participants, consisting of 15 Qatari and 123 Non-Qatari women. The average age and standard deviation was 31.492, 5.880 years for GDM and 30.453, 5.792 for control, respectively. Additionally, the average weight was recorded as 92.295 KGs for the GDM group and 83.946 KGs for the control group. Tables 3, 4 summarize the baseline statistics of the cohort from QBB.

**Table 3:** Baseline Statistics of Participants

Feature	Mean (GDM)	STD (GDM)	Mean (Control)	STD (Control)	p-value
Chronological Age (years)	31.492	5.880	30.453	5.792	0.1496
Pregnancy Age (weeks)	16.046	3.952	16.693	4.299	0.181
Current weight (kg)	92.295	116.970	83.946	107.923	0.331
PrePregnancy Weight (kg)	89.745	117.288	93.084	151.511	0.443
Weight Difference (kg)	30.044	231.749	11.129	108.364	0.0862

**Table 4:** History of High Glucose/Diabetes Statistics with Nationality

Feature	Yes	No
Qatari	15	123
Family History of Diabetes	138	1
History of High Glucose Level/Diabetes	32	106

### Feature subset selection and their correlation

**Fig 2.**
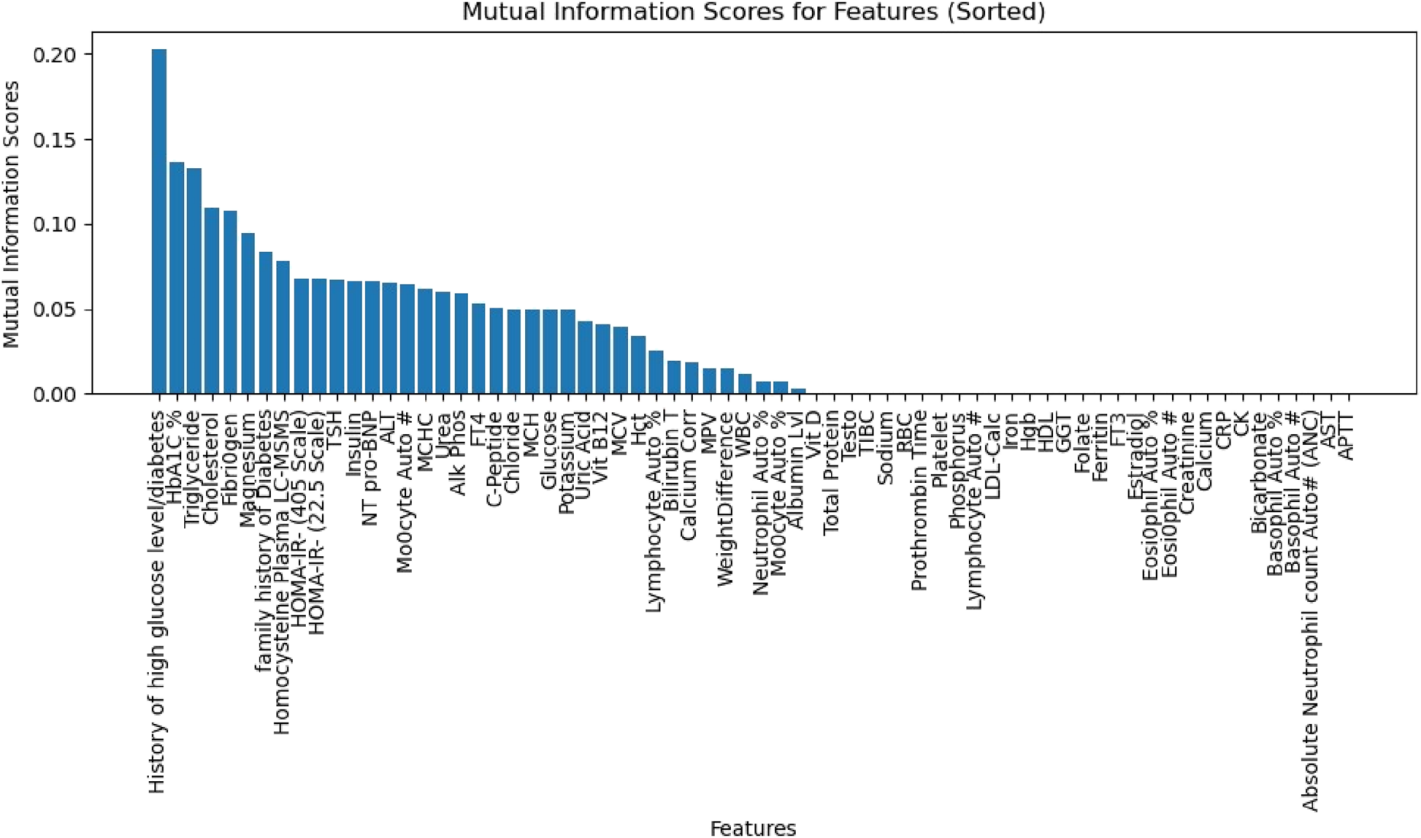
MI scores for all the variables in our dataset

**Fig 3.**
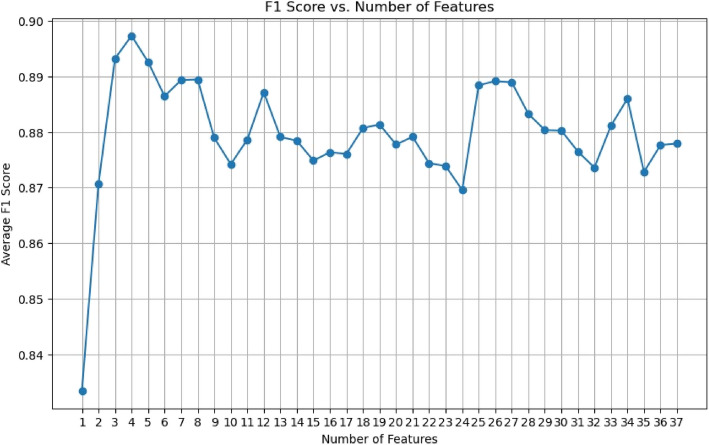
Model F1 score vs number of selected features

We applied a two-step process to select the most essential features from the available dataset. In the first step, we selected a group of features based on MI. Then, in the second step, we further reduced the feature subset by applying F1-scoring based filtering technique. Figure 2 shows the MI Scores for all the features of our dataset. We selected the top 37 features from this list with an MI score above zero. For the rest of the variables, MI scores were too low to be considered. Next, we trained the ML model and plotted the average F1 score for the selected 37 features (Figure 3). By systematically iterating through top-ranked features based on F1-score, we selected the top 26 not the top 4 features to avoid overfitting, the features are: ’History of high glucose level/diabetes’, ’HbA1C %’, ’Triglyceride’, ’Cholesterol’, ’Fibriogen’, ’Magnesium’, ’family history of Diabetes’, ’Homocysteine Plasma LC-MSMS’, ’HOMA-IR- (405 Scale)’, ’HOMA-IR- (22.5 Scale)’, ’TSH’, ’Insulin’, ’NT pro-BNP’, ’ALT’, ’Monocyte Auto ’, ’MCHC’, ’Urea’, ’Alk Phos’, ’FT4’, ’C-Peptide’, ’Chloride’, ’MCH’, ’Glucose’, ’Potassium’, ’Uric Acid’, and ’Vit B12’.

**Fig 4.**
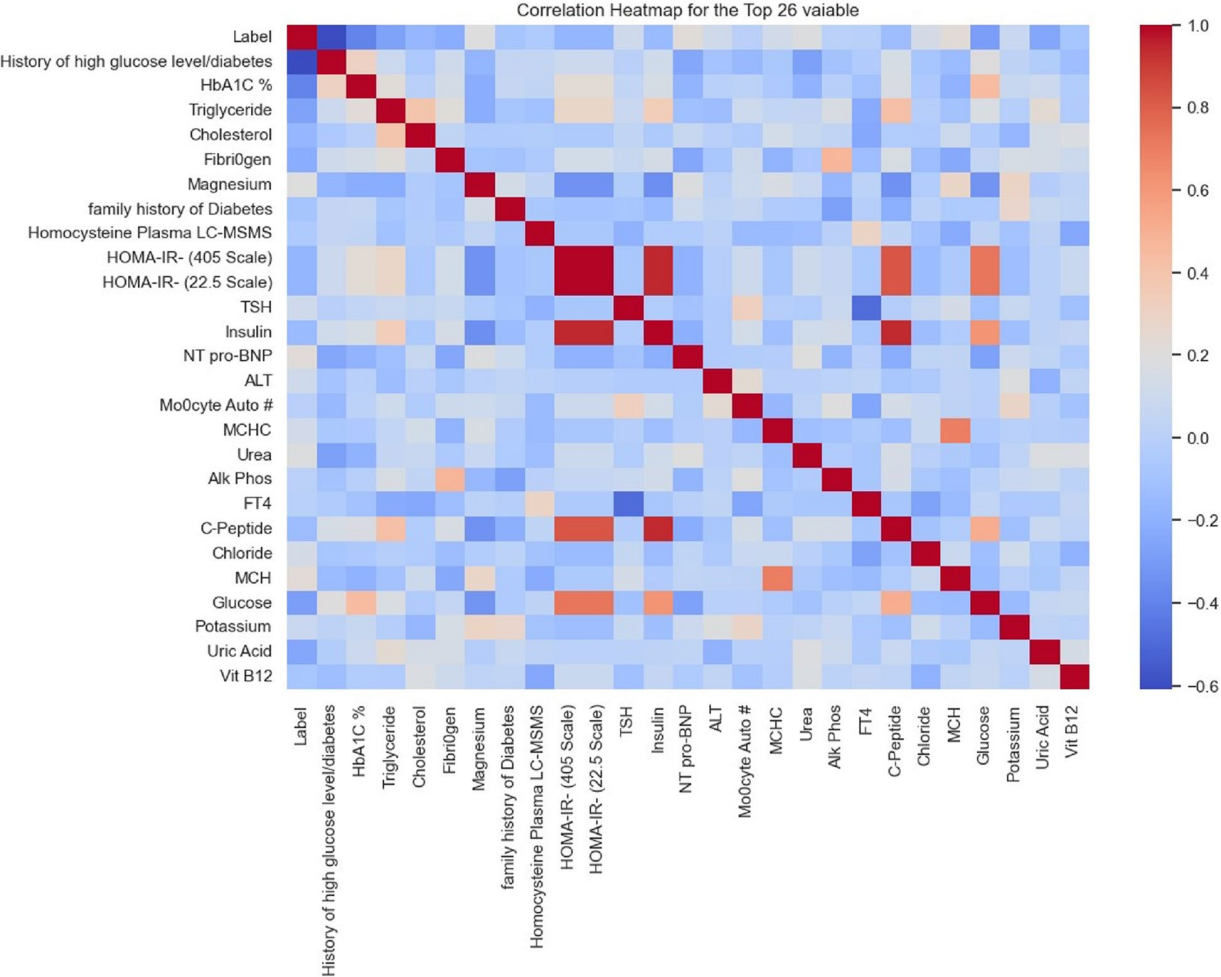
Correlation of the selected features

The correlation of these 26 features are shown in Figure 4.

### Performance of machine learning model

We tested our model using the 37 features selected based on the MI score (Table 5) and 26 features based on the F1 score (Table 6). Considering the 26 features improved the model performance, with the best model achieving an average accuracy of 88.8% (Table 6). This metric indicates the proportion of correct classification cases, reflecting the general correctness of the model forecast.

**Table 5:** Model Results using 37 variables with an MI score grater than 0

Model	Sn (Recall)	Sp	Acc	F1-score	Precision
RandomForestClassifier	78.40%	77.80%	77.89%	78.64%	79.82%
GradientBoostingClassifier	81.10%	76.99%	78.89%	80.01%	80.05%
AdaBoostClassifier	81.77%	75.14%	78.33%	79.77%	78.81%
DecisionTreeClassifier	75.34%	78.36%	76.56%	76.99%	79.61%
LogisticRegression	77.05%	73.39%	75.83%	75.91%	75.60%
SVC	78.10%	74.43%	76.17%	77.42%	78.12%
GaussianNB	81.64%	69.66%	76.44%	78.03%	75.92%
KNeighborsClassifier	79.92%	58.55%	71.00%	71.63%	65.43%
CatBoostClassifier	86.14%	73.99%	80.50%	81.21%	77.69%
XGBClassifier	79.41%	77.76%	78.39%	79.21%	80.04%
LGBMClassifier	70.63%	66.51%	69.94%	69.17%	68.96%
Stacking	90.19%	83.50%	87.22%	87.99%	86.16%

**Table 6:** Model Results using the top 26 variables selected from 37 variables producing the highest F1-score

Model	Sn (Recall)	Sp	Acc	F1-score	Precision
RandomForestClassifier	81.72%	78.51%	80.22%	81.08%	81.55%
GradientBoostingClassifier	82.85%	77.69%	80.11%	81.31%	80.83%
AdaBoostClassifier	85.67%	78.24%	81.94%	83.27%	82.15%
DecisionTreeClassifier	79.16%	78.51%	78.78%	79.61%	81.35%
LogisticRegression	80.55%	77.44%	79.22%	79.18%	78.53%
SVC	80.34%	75.97%	78.06%	79.29%	79.58%
GaussianNB	82.81%	70.23%	77.28%	78.78%	76.53%
KNeighborsClassifier	88.29%	67.25%	78.39%	81.14%	75.80%
CatBoostClassifier	89.22%	73.62%	81.56%	83.45%	79.34%
XGBClassifier	78.62%	79.04%	78.61%	79.18%	80.93%
LGBMClassifier	73.85%	71.92%	73.39%	73.38%	73.82%
Stacking	92.13%	84.94%	88.83%	89.56%	87.34%

We also evaluated the model’s performance using other important metrics such as sensitivity (recall), specificity, precision, and F1-score, in addition to accuracy. These metrics provide a more thorough assessment of the model’s effectiveness and uncover potential flaws.

- Sensitivity (Recall): This metric measures the percentage of actual positive cases (GDM) that are correctly identified by the model. The high sensitivity of our model indicates that it is effective in capturing true positive cases and minimizing false negatives.
- Specificity: The proportion of actual negative cases (non-GDM) that are correctly identified is measured by this metric. High specificity indicates that the model is effective in avoiding false positives, which is important in a clinical setting to prevent unnecessary interventions.
- Precision: Precision measures the percentage of positive identifications that are actually correct. High accuracy ensures the model’s accuracy in predicting positive outcomes, reducing the risk of erroneous predictions.
- F1-score: A balanced evaluation of the model’s performance can be provided by the F1-score. It’s especially useful when the data has a mixed grouping, as it takes into account both true positives and false negatives.

The average precision is calculated at 87.3%, a measure of the model’s ability to prevent false positives (Table 6).This metric is particularly relevant in medical contexts since misclassifying a healthy case as positive (false positive) should be minimized. The average recall rate is 92.1%, which measures the model’s effectiveness in capturing true positives. In order to ensure that a significant proportion of the actual positive cases are correctly identified, high recall is essential for medical diagnosis. The model introduced a well performed average F1-score of 89.6%. This measure provides a balanced assessment of the model’s overall performance, considering accuracy and recall. The effectiveness of the developed model in detecting gestational diabetes, based on first-trimester data, is highlighted by these results. The high average recall indicates a robust ability to capture positive cases, while the high precision and F1 score prove a balanced performance in minimizing false positives. In support of the model’s potential to be applied in real-world scenarios, reported metrics demonstrate its reliability and accuracy as an ealry gestational diabetes predictor.

### Evaluating the Model’s Performance using a set of Random Seeds

**Fig 5.**
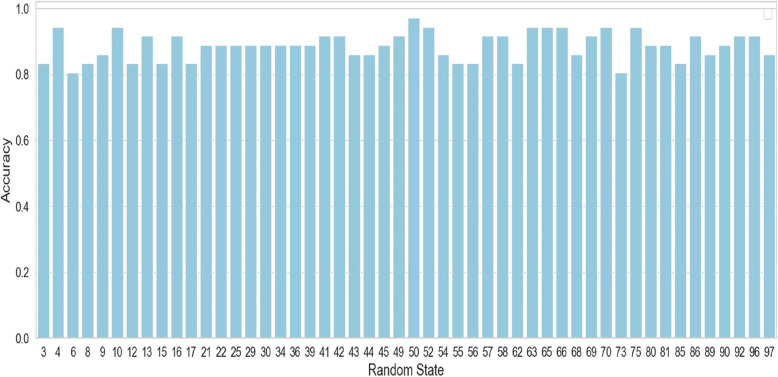
Model Accuracy Variation among Random seeds

Random seed plays a vital role in initializing model parameters, influencing the next stage of training, and helping to assess the robustness and reliability of research findings. We have systematically investigated this influence in the initial phase of model training by intentionally varying random seeds to gain a more comprehensive understanding. This intentional variation allowed a thorough assessment of its apparent impact on primary performance metrics such as precision, recall, accuracy, and F1- score in multiple trials. Figure 5 highlights the change in accuracy over a set of random seeds, which we used for generating the average evaluation metrics for our predictor.

For instance, with a particular randomly selected seed (such as 52) an important observation was detected resulting in an accuracy of 100%. While this may suggest superior model performance in the training and testing phase, it is crucial to note that such perfection may not guarantee effective handling of real-life data randomness. Real-world data inherently differ from training data and emphasize the need for a delicate understanding of the model’s adaptability beyond control environments.

### Clinical Biomarkers identified from the model

Based on our analysis, we identified 26 biomarkers that contribute the most to our model for the detection of GDM. Table 7 highlights their basic statistics in the GDM group as well as in the control group. Out of these 26 variables, 11 were statistically significant.

**Table 7:** Most Important 26 Biomarkers Identified by the Model

Feature	Case-Mean	Case-STDdev	Control-Mean	Control-STDdev	p-value
History of high glucose level/diabetes	0.508	0.504	0.000	0.000	0.0000
HbA1C %	5.356	0.498	5.005	0.261	0.0000
Glucose	4.790	1.062	4.271	0.532	0.0001
MCH	27.517	2.428	28.729	2.060	0.0009
NT pro-BNP	46.681	33.107	60.274	36.233	0.0120
HOMA-IR (22.5 Scale)	6.486	11.355	3.233	5.439	0.0148
HOMA-IR (405 Scale)	0.360	0.631	0.180	0.302	0.0148
Insulin	25.421	32.277	16.047	23.941	0.0263
Magnesium	0.726	0.057	0.745	0.060	0.0299
Uric Acid	189.730	40.612	176.887	43.085	0.0378
C-Peptide	3.035	2.037	2.436	1.954	0.0403
Triglyceride	1.746	0.600	1.576	0.663	0.0598
Urea	2.337	0.569	2.489	0.627	0.0697
Chloride	101.127	1.680	101.653	2.413	0.0734
Fibrinogen	4.384	0.584	4.218	0.805	0.0880
MCHC	33.716	1.140	33.971	1.081	0.0904
ALT	11.865	6.613	13.823	10.667	0.1038
Family History of Diabetes	1.000	0.000	0.987	0.115	0.1807
Vit B12	242.581	110.993	230.460	96.509	0.2469
TSH	1.955	1.170	2.088	1.208	0.2577
Potassium	4.046	0.236	4.065	0.225	0.3122
Alk Phos	59.524	16.419	58.173	18.640	0.3276
Cholesterol	5.410	1.025	5.355	1.216	0.3900
FT4	13.798	2.722	13.753	1.549	0.4509
Homocysteine Plasma LC-MSMS	5.919	1.763	5.951	1.258	0.4513
Monocyte Auto	0.507	0.162	0.506	0.137	0.480

**Fig 6.**
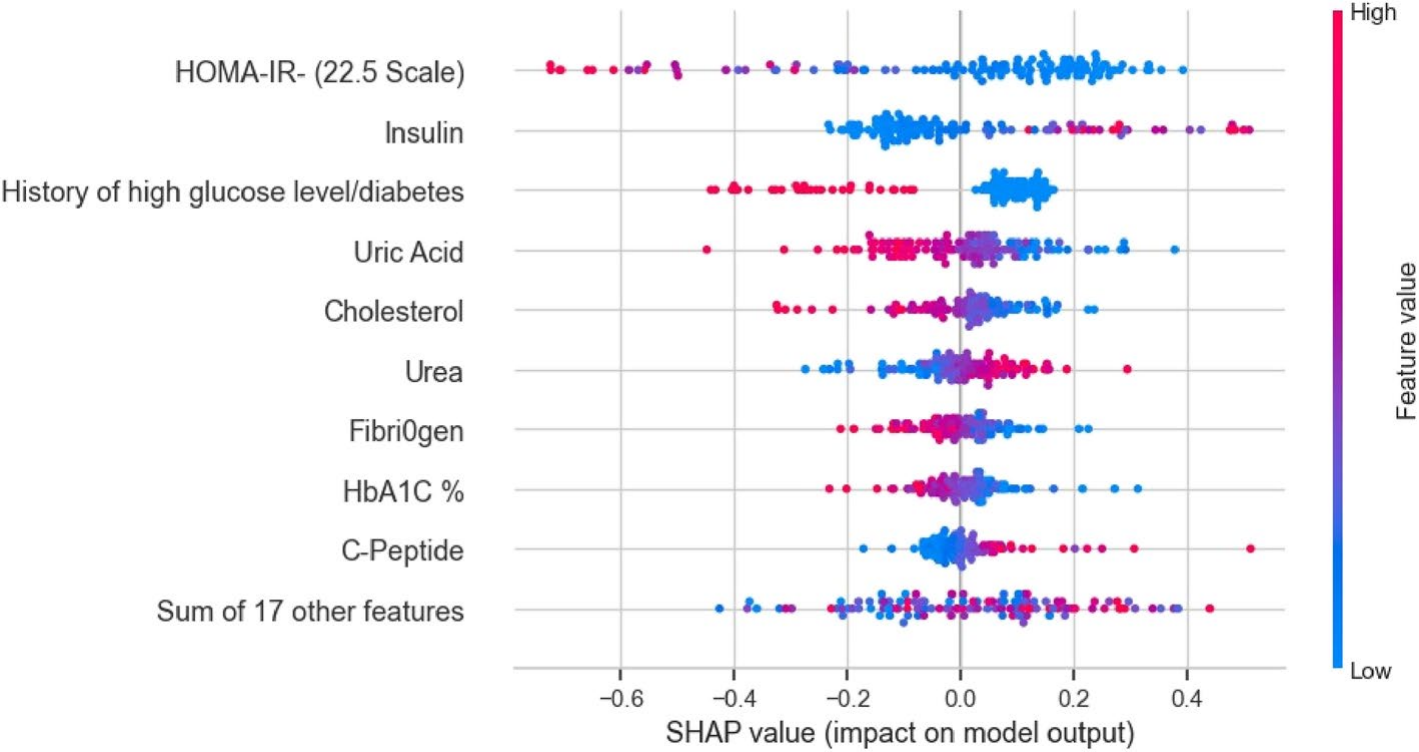
SHAP Plot for the selected 26 variables

HOMA-IR(22.5 Scale) identified with the highest impact on the model prediction To explain the importance of the identified clinical markers (Table 7), we also used the SHAP plot (Figure 6) to highlight the relative importance of the selected features of the proposed model. The SHAP plot shows that HOMA-IR(22.5 Scale) was the most dominant feature for identifying the GDM group from the control group. The second and third most dominant markers were insulin and history of diabetes or high glucose levels.Uric Acid and other features were also identified as a potential clinical biomarker from our model.

### Role of potential confounding variables

In this study, we included additional confounding variables such maternal age, dietary habits, and lifestyle factors, going beyond conventional analyses based only on medical or laboratory data. We divided the dataset into pertinent subgroups and used the top 26 characteristics to train the model in order to gain a better understanding of how these variables affected model performance. This method demonstrated notable variations in the model’s performance among subgroups. Maternal age likely influences the model’s predictions, for instance, the younger group (less than or equal 30 years) had a higher AUC of 92.1% when stratified by maternal age, compared to 91.2% for the older group (more than 30 years). Dietary practices and levels of physical activity also revealed similar patterns. With the high-level of physical activity group achieving the greatest AUC of 95.6% and the low-level of physical activity group displaying a lower AUC of 89.7%. With the high-sugar intake group achieving the greatest AUC of 85.2% and the low-sugar intake group displaying a lower AUC of 93.6%. These findings highlight how important confounding variables are in influencing the model’s predicted results.

Stratification, however, introduces an inherent challenge of data imbalance, as subgroups often have unequal sample sizes. The validity of traditional measurements like accuracy or precision may be compromised by this mismatch. Given its resilience in managing unbalanced data and its capacity to evaluate the model’s discriminatory ability in a comprehensive manner, AUC was given priority as the main assessment metric in order to solve this. The significant variations in AUC between subgroups highlight how crucial it is to take confounding variables like maternal age, dietary habits, and lifestyle factors into consideration. These results show that adding these factors and stratifying according to them improves the model’s dependability and guarantees that it captures significant variability across a range of populations.

## Discussion

This study was conducted for early prediction of GDM in the Qatari population using only first-trimester clinical data and ML-based techniques. It is among the first studies to be conducted in Qatar for early detection of GDM using ML models. Our ML model detected GDM from the control group with a high accuracy of 88.8%. In addition, we identified the clinical biomarkers that contributed the most to the model for early detection of GDM, of which seven biomarkers were statistically significant. We clarified the contribution of the most prominent features for the early detection of GDM based on the SHAP method. Additionally, our model might positively impact the management of GDM. Lifestyle interventions are usually used as the primary method of managing GDM patients [41]. However, pharmacological treatments, such as insulin and Metforminare also necessary in some cases. Treatment with insulin is preferred over metformin to lower blood glucose levels. At the same time, metformin is considered a secondary treatment for GDM, since the medication crosses the placenta, and its long-term effect on the fetus is still unclear [42, 43]. Early and proper treatment of GDM might reduce the risk of any potential complications in both the mother and the fetus. Therefore, early prediction of GDM using ML-based techniques will be of great importance for the early treatment and prevention of GDM.

We identified HOMA-IR using the SHAP method, which had the highest ability to differentiate the GDM group from the control group, in addition to other clinical biomarkers such as history of diabetes and NT pro-BNP. As proposed by some of the previously published models that predict risk factors asso- ciated with GDM among pregnant women, a history of high glucose level/diabetes, HOMA-IR [44], MPV and Prothrombin time, and many other factors were related to the risk of GDM [17, 45, 46]. To identify the risk factors, we collected first-trimester data from a cohort of pregnant women. As a result, we identified a significant difference in the pre-pregnancy weight between the GDM and the control women. Most studies focused on the elevated pre- pregnancy body mass index, which measures body fat based on weight and height, and weight increase during pregnancy as a risk factor for GDM [47–49]. At the same time, others concentrated on studying the effect of pre-pregnancy weight on early GDM development, were Deshpande et al. discovered a positive association between pre-pregnancy weight and the risk of GDM [50]. These findings further support the notion that higher pre-pregnancy weight may predispose women to develop GDM during pregnancy. Furthermore, Deshpande et al. revealed a relationship between pregnant women’s body weight and HOMAIR, where the Homeostasis model assessment of insulin resistance (HOMA-IR) is a method to quantify insulin resistance. A higher HOMA-IR level due to changes in maternal hormones during pregnancy means a higher insulin resistance [50]. Insulin resistance plays a crucial role in the development of GDM, where insulin resistance causes impaired normal glucose metabolism and contributes to hyperglycemia during pregnancy [51]. Thus, Deshpande et al. identified HOMA-IR as a risk factor for GDM in addition to the relationship between HOMA-IR and weight [50]. Our study corroborates these findings, highlighting HOMA-IR as one of the most dominant features associated with the risk of GDM. Furthermore, it shows a clear rela- tionship between the history of diabetes, insulin levels, and HOMA-IR, discovered as the most dominant biomarkers when we applied the Shapley additive explanations (SHAP) method to clarify features’ contribution and importance to the predicted GDM risk. This relationship might be explained by understanding the pathophysiology of GDM. Insulin resistance, de- fined as inadequate glucose uptake by peripheral tissues, induces pancreatic -cells to produce more insulin to lower blood glucose levels to compensate for the resistance, which burdens the -cells with more stress and exacerbates their dysfunction. In most cases, pancreatic -cells impairments exist even before pregnancy, which indicates a history of diabetes in the patients [51].

B-type natriuretic peptide (BNP) is a hormone secreted in response to various circum- stances when the pressure increases the tension on ventricle cardiomyocytes. The N-terminal part of BNP, known as NT-proBNP, is usually a biomarker of heart failure. In 2016, NT- proBNP was shown to be a valuable diagnostic marker of preeclampsia and gestational hypertension. However, this was not the case in GDM, where Sadlecka et al. and Andreas et al. found no significant difference in NT-proBNP levels between women with and without GDM [52, 53]. Our findings indicated that NT pro-BNP is a potential clinical biomarker of GDM, which conflicts with the previous studies. The differences in the study population might explain this conflict. For example, Sadlecka et al. included patients with singleton pregnancies suffering from different complications, such as preeclampsia and gestational hypertension. However, further studies are needed to uncover the relationship between NT-proBNP levels and GDM, which could provide more insights into the utility of NT-proBNP as a diagnostic marker in GDM. One of the other risk factors that showed disagreement with previous studies is cholesterol. Changes in lipid metabolism are a phenomenon that usually occurs during pregnancy. Thus, LDL and total cholesterol increase during pregnancy. In this study, high cholesterol level was associated with the risk of GDM. However, a previous study revealed a slight increase in total cholesterol and LDL-C levels among women with GDM compared to matched controls and no significant association with the risk of GDM. Large cohort studies are needed to confirm the association between cholesterol levels and the risk of GDM [54]. Furthermore, pregnancy induces substantial changes in various functions, such as the thyroid gland’s metabolic function. For example, the size of the thyroid gland increases greatly to produce enough thyroid hormones (T4 and T3) to manage the increasing demand during pregnancy. These thyroid hormones are vital in glucose metabolism and might be associated with GDM. As a result, one of the previous studies discovered a positive correlation between FT3 and GDM [55], which agrees with our finding. Moreover, we observed a significant difference in magnesium levels between the cases and controls and a noticeable association with GDM. This finding is confirmed by a previous study where RBC-Mg levels were remarkably lower in the GDM group than in the controls [56]. Finally, we found that urea is associated with the risk of GDM; however, previous experimental studies highlighted only urea nitrogen’s association with GDM [57]. Machine learning models for GDM prediction have been previously investigated in several studies, including Zhang et al. [19], Liu et al. [23], Li et al. [24], Watanabe et al. [31], and Xiong et al. [17]. Our findings are in line with those studies since we identified the following potential biomarkers for early GDM prediction: history of high glucose level/diabetes, Insulin, Cholesterol, and LDL-C.

Overall, we can conclude that insulin, NT pro-BNP, cholesterol, MCHC, FT3, prothrombin time are potential clinical biomarkers for early GDM detection according to our analysis. Furthermore, HOMA-IR score (which combines insulin and glucose level) and history of diabetes are among the two most influential indicators for early GDM detection. Further validation on larger cohorts of GDM patients is required to confirm the accuracy of our models for the early detection of GDM during the first trimester of pregnancy.

To ensure the practical applicability and benefit of our work in clinical settings, we propose multiple guidelines for its implementation. Patient record should be entered digitally into EHR so that analysis can be done automatically. Automated data extraction from EHRs will improve workflow efficiency and decrease errors in human. The AI model implementation in a clinical setting may require collaboration between endocrinologists, obstetricians, data scientists, and IT professionals. To ensures that the model is fully utilized and integrated in an effective manner, their seamless integration is required. A high predictive accuracy of 91.3 percent ensures reliable early detection of GDM, minimizing false positives and negatives. In clinical settings where accurate diagnosis is important, this level of precision is critical. In order to apply the model result effectively, healthcare professionals should receive adequate training on the usage of AI models. Understanding the role of AI as a supporting tool will help them to make wise therapeutic decisions.

There are a few limitations of this study. One primary limitation is that model performance always depends on the quality and diversity of the training data. We work on a relatively small dataset, therefore, we need to improve and validate the model on larger cohort to confirm its robustness and generalizability. Additionally, the model depends upon biomarkers which will require blood sample collection followed by lab testing. This is relatively time-consuming and expensive process. Therefore, this model might not be applicable in resource-limited healthcare setup.

## Data Availability

Data used in this research can be accessed upon the approval from QBB. Please contact takepart@qatarbiobank.org.qa for data access.
